# Long noncoding RNA MIAT promotes non-small cell lung cancer proliferation and metastasis through MMP9 activation

**DOI:** 10.18632/oncotarget.21465

**Published:** 2017-10-03

**Authors:** I-Lu Lai, Chin-An Yang, Pei-Chin Lin, Wen-Ling Chan, Ya-Ting Lee, Ju-Chen Yen, Ya-Sian Chang, Jan-Gowth Chang

**Affiliations:** ^1^ Epigenome Research Center, China Medical University Hospital, Taichung, Taiwan; ^2^ Department of Laboratory Medicine, China Medical University Hospital, Taichung, Taiwan; ^3^ Division of General Pediatrics, Children's Hospital of China Medical University, Taichung, Taiwan; ^4^ College of Medicine, China Medical University, Taichung, Taiwan; ^5^ Graduate Institute of Clinical Medicine, Kaohsiung Medical University, Kaohsiung, Taiwan; ^6^ Department of Pediatrics, School of Medicine, College of Medicine, Kaohsiung Medical University, Kaohsiung, Taiwan; ^7^ Division of Hematology and Oncology, Department of Pediatrics, Kaohsiung Medical University, Kaohsiung, Taiwan; ^8^ Department of Bioinformatics and Medical Enginerring, Asia University, Taichung, Taiwan

**Keywords:** MIAT, long noncoding RNA (lncRNA), non-small cell lung cancer (NSCLC), mixed-lineage leukemia (MLL), matrix metallopeptidase 9 (MMP9)

## Abstract

Long noncoding RNAs (lncRNAs) play crucial roles in carcinogenesis. Myocardial infarction-associated transcript (MIAT), originally isolated as a candidate gene for myocardial infarction, has been found to act as an oncogene in chronic lymphocytic leukaemias and neuroendocrine prostate cancer (NEPC); however, little is known about its expression pattern, biological function, and underlying mechanism in non-small cell lung cancer (NSCLC). In this study, we observed that MIAT expression was upregulated in NSCLC, and its overexpression was associated with advanced tumor stage. Moreover, MIAT knockdown decreased cell proliferation, migration, invasion, and cell cycle arrested in G1 phase. Mechanistic investigation revealed that MIAT could interact with histone methyltransferase mixed-lineage leukemia (MLL). MIAT silencing impeded the binding of MLL on the matrix metalloproteinase 9 (MMP9) promoter region and epigenetically reduced MMP9 transcriptional activity. Overall, our findings suggest that MIAT expression is associated with NSCLC and may be one of the critical targets in progression and metastasis in NSCLC.

## INTRODUCTION

Lung cancer is the leading cause of cancer-related deaths worldwide, with non-small cell lung cancer (NSCLC) accounting for approximately 85% of all cases [1]. Most NSCLC patients are diagnosed at an advanced stage and have a 5-year survival rate of less than 20% [1, 2] because of their advanced stage diagnoses [3]. Lack of early diagnosis markers and high potential for the invasion ability of NSCLC are challenging for NSCLC therapy. Hence, the molecular mechanisms involved in the development and progression of NSCLC must be investigated.

Long noncoding RNAs (lncRNAs), defined as a class of noncoding RNA with a length of more than 200 nucleotides, have critical roles in the gene expression regulation [4], epigenetic control [5], chromatin structure [6, 7], development process, genomic imprinting, and pluripotency of embryonic stem cells [6, 8, 9]. In addition, dysregulation of lncRNAs has been reported to play a vital role in the carcinogenesis, disease progression, and metastasis of human cancers [6, 7, 10–12]. Some lncRNAs such as H19, HOTAIR, ANRIL, MALAT1, and SCAL1 [13–15] have been reported to be associated with the development and progression of lung cancers. However, the roles of lncRNAs in NSCLC development and metastasis remain largely unknown. Hence, the identification of lung cancer-associated lncRNAs and the investigation of their molecular and biological functions in lung cancers are vital.

Myocardial infarction-associated transcript (MIAT) is one of the noncoding RNAs first identified as an lncRNA in 2006 [16]. MIAT is involved in various cellular processes, including myocardial infarction [16, 17], microvascular dysfunction [18], paranoid schizophrenia [19], nuclear body formation [20], and neurogenic commitment [21]. Because MIAT physically interacts with SF1 splicing factor, it is supposed to be involved in RNA splicing and regulating gene expression [22]. Recent studies have demonstrated that MIAT constitutes a loop with Oct4 in malignant mature B cells and is essential for cell survival [23]. MIAT is also upregulated and interacts with the polycomb in neuroendocrine prostate cancer to participate in tumorigenesis [24]. However, the expression pattern, biological function, and underlying mechanism of MIAT in NSCLC are still unclear.

In the present study, we investigated the potential mechanisms of MIAT in NSCLC progression. We observed that MIAT was upregulated and played a role in the advanced pathological stage. Moreover, our data revealed that MIAT could interact with MLL and epigenetically activate MMP9 to facilitate cell proliferation, migration, and invasion in NSCLC.

## RESULTS

### MIAT expression was upregulated and correlated with advanced tumor stage

**Figure 1:**
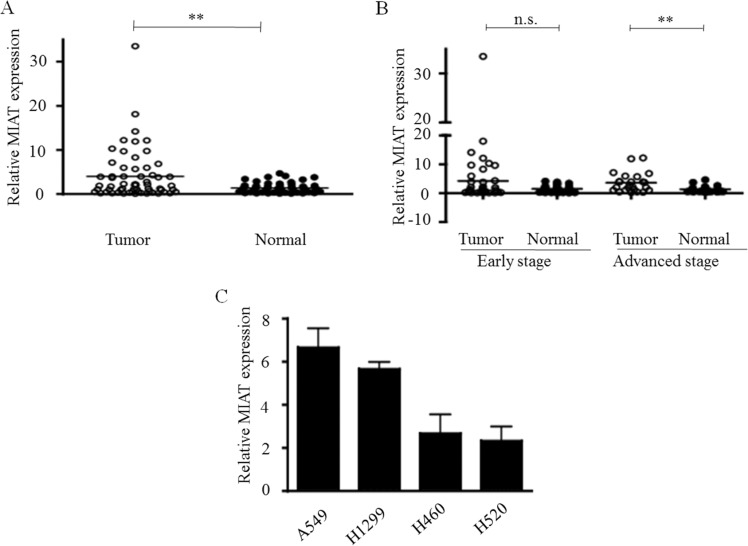
Relative MIAT expression in NSCLC and its clinical significance (**A**) MIAT was overexpressed in primary human NSCLC compared with adjacent normal tissues (*n* = 60 for each group). (**B**) Higher MIAT expression levels in NSCLC was significantly correlated with advanced tumor stages. (**C**) The relative expressions of MIAT in NSCLC cell lines as determined by real-time PCR. ^**^p < 0.01, n.s. means no significance. Statistical analysis was conducted using student *t*-test

To explore whether MIAT played a role in carcinogenesis, we first profiled the expression of MIAT in 60 pairs of NSCLC tissues (30 paired of adenocarcinoma and 30 paired of squamous) and paired adjacent non-tumor tissues. The qPCR data indicated that the expression level of MIAT in tumor tissues was significantly higher than that in the corresponding non-tumor tissues (mean dCT of tumor vs. normal tissue: 2.95 vs. 3.71, *p* = 0.0014; Figure 1A). Furthermore, we analysed the association between *MIAT* gene expression and the clinical stage of NSCLC and the state of metastasis. *MIAT* upregulation in the tumor tissues was associated with an advanced stage (stages III, IV, *n* = 24, *p* = 0.001) but not early stage cancer (stages I and II, *n* = 36, *p* = 0.09; Figure 1B). Next we tested the MIAT expression in NSCLC cell lines, including A549, H1299, H460, and H520. Among these cell lines, MIAT was relative higher expressed in A549 and H1299 (Figure 1C); thus, we chose A549 and H1299 cells to perform the following experiments. Moreover, to investigate the clinical significances of MIAT, we evaluated the correlation between MIAT level and clinicopathological factors. Results revealed that MIAT levels were correlated with tumor size (*p* = 0.0035), TMN stage (*p* = 0.001), and lymph node metastasis (*p* = 0.0185) in NSCLC. Nevertheless, MIAT levels were not associated with age (*p* = 1.000) or gender (*p* = 0.0581) (Table 1). These results indicated that upregulated expression of MIAT might play a role in NSCLC tumorigenesis.

**Table 1:** MIAT expression and clinicopathological factors in NSCLC patients (*n* = 60)

Parameter	*N*	Relative MIAT expression		*p*-value
		Low	High	
Age (year)				1.000
≤ 65	34	20	14	
> 65	26	16	10	
Gender				
Male	37	26	11	0.0581
Female	23	10	13	
Tumor size (maximum diameters)				
≤ 3 cm	32	25	7	0.0035
> 3 cm	28	11	17	
Lymph node metastasis				
N1	26	11	15	0.0185
N0	34	25	9	
TMN stage				
I–II	36	28	8	0.0010
III–IV	24	8	16	

*P* values when expression levels were compared using Fisher's exact test.

### Knockdown of MIAT impaired lung cancer cells proliferation and cell cycles arrest *in vitro*

**Figure 2:**
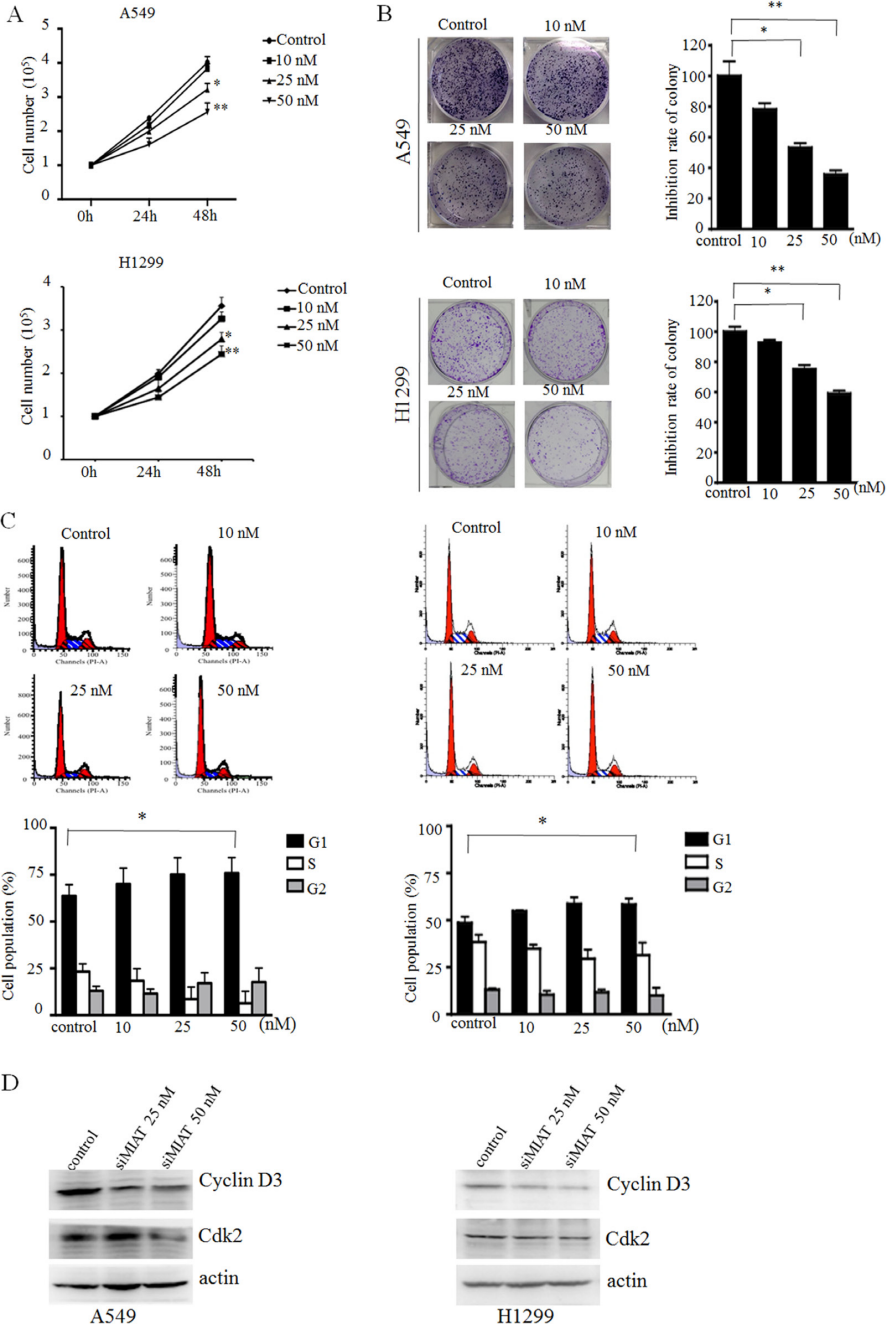
MIAT knockdown represses cell proliferation and cell cycle progression *in vitro* (**A**) Cell counting assay indicated that MIAT knockdown repressed A549 and H1299 cell proliferations. (**B**) Colony formation experiment demonstrated that MIAT knockdown represses A549 and H1299 cell proliferations. (**C**) Representative images and quantification of the flow cytometry analyses of A549 (left) and H1299 (right) after transfection. Cell cycle analyses revealed that MIAT influences A549 and H1299 cell proliferations by regulating their cell cycle. The bar chart shows the percentage of cells in G0/G1, S, and G2/M phase. (**D**) G1 arrest cell cycle markers were analysed by western blot in siMIAT-transfected A549 cells (left) and siMIAT-transfected H1299 (right). Values are represented as mean ± SD from three independent experiments. All experiments were performed in triplicate. ^*^p < 0.05, ^**^p < 0.01. Statistical analysis was conducted using student *t*-test.

Because the overexpression of MIAT was significantly associated with progression in NSCLC patients, we further modulated MIAT expression to examine whether MIAT regulated the proliferation of A549 and H1299 cells. A cell counting assay revealed that cell growth rate of A549 and H1299 were dose-dependently inhibited with siMIAT compared with the control (Figure 2A). Colony formation assay data also revealed that clonogenic survival were inhibited in si-MIAT-treated A549 and H1299 cells (Figure 2B). To further examine whether the effect of MIAT on proliferation reflected cell cycle arrest, cell cycle progression was analysed using flow cytometry analysis. The results indicate that MIAT knockdown retarded the G1/S transition in si-MIAT A549 and H1299 cells (Figure 2C). We then performed Western blot and found that knockdown of MIAT would decrease the expressions of cyclin D3 and cdk2 in A549 and H1299 cells (Figure 2D). These data indicated that MIAT could promote the proliferation phenotype of NSCLC cells.

### MIAT silencing impaired cell migration and invasion *in vitro*

**Figure 3:**
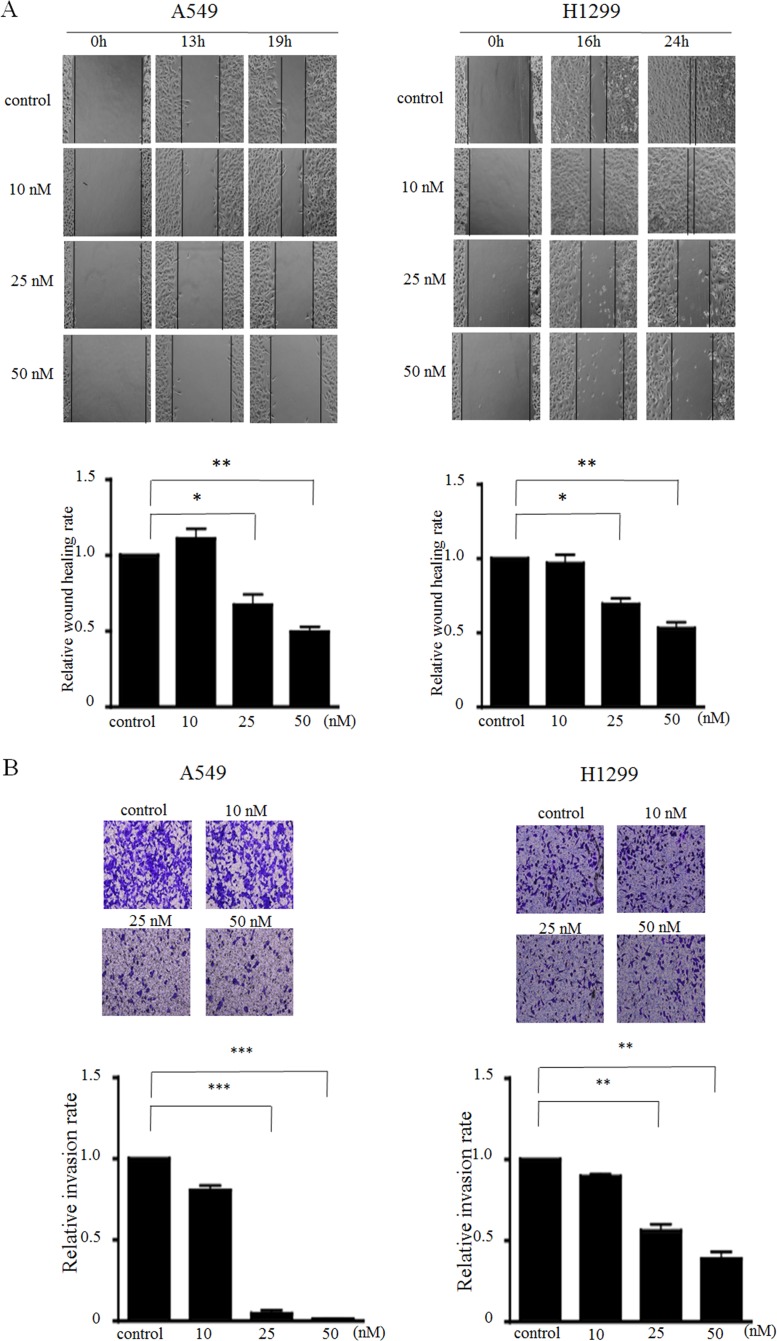
MIAT knockdown inhibits cell migration and invasion *in vitro* (**A**) Wound scratch assays were performed to analyse the migration efficiencies of siMIAT-transfected A549 (left) and siMIAT-transfected H1299 (right). The statistical analysis of the inhibition rates of A549 and H1299 were performed at 19 h and 24 h, respectively. (**B**) Transwell invasion assay was performed to determine the invasion ability for control and siMIAT-transfected A549 cells (left) and siMIAT-transfected H1299 (right). Data are presented as mean ± SD. ^*^p < 0.05, ^**^p < 0.01, ^***^p < 0.001. Statistical analysis was conducted using student *t*-test.

Next, we explored the efficiency of MIAT on migration and invasion in A549 and H1299 cells. The wound healing scratch assay revealed that the ratio of the recovered region were decreased in MIAT knockdown A549 and H1299 cells compared with the control (Figure 3A). Furthermore, a matrigel transwell assay demonstrated that decreasing MIAT expression could dose-dependently impede the invasion abilities of A549 and H1299 cells (Figure 3B) compared with control. These results indicate that knockdown of MIAT expression retarded cell migration and invasion motility in NSCLC cells.

### MIAT silencing impaired A549 cell migration and invasion *in vivo*

**Figure 4:**
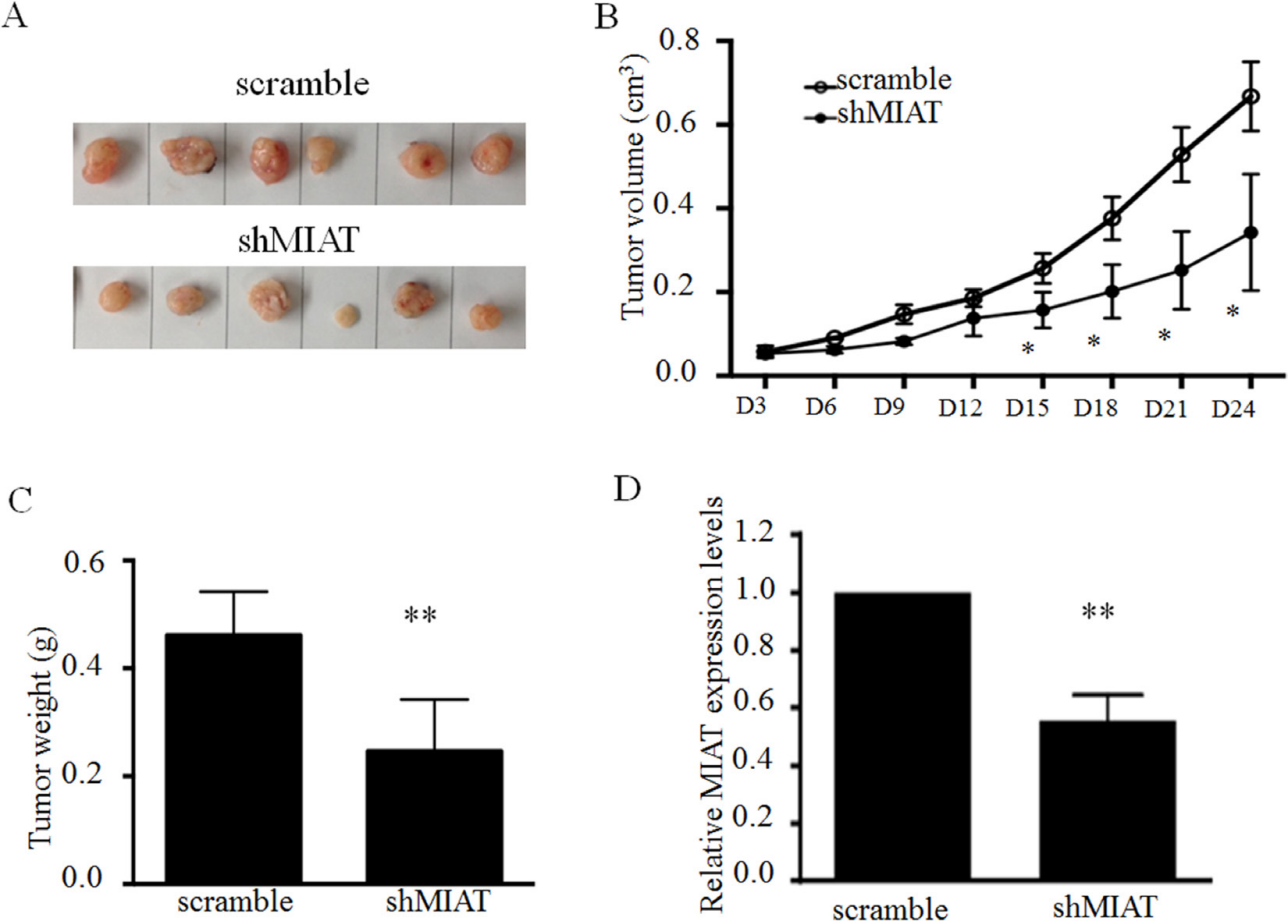
MIAT knockdown represses tumor growth *in vivo* (**A**) Representative image of tumors isolated from nude mice. (**B**) Tumor growth curves. (**C**) Tumor weights are represented. (**D**) QPCR analysis of MIAT expression in tumor tissues formed from A549/scramble and A549/shMIAT. Data are presented as mean ± SD. ^*^p < 0.05, ^**^p < 0.01. Statistical analysis was conducted using student *t*-test.

To validate the oncogenic efficiency of MIAT *in vivo*, A549 cells stably transfected with shMIAT or scramble were subcutaneously inoculated into the left flank (stable shMIAT cells) and right flank (scramble cells) of BALB/c athymic male nude mouse, respectively (*n* = 6). After 24 days, the tumors formed in the shMIAT group were substantially smaller than those in the scramble group (Figure 4A and 4B). Moreover, the tumor weight at the end of the experiment was lower in the shMIAT group (0.466 ± 0.021 g) compared with that in the scramble group (0.333 ± 0.051 g) (Figure 4C). QPCR analysis confirmed that the MIAT levels were lower in shMIAT tumor tissues than in scramble tumor tissues (Figure 4D). These findings indicate that *MIAT* knockdown would decelerate tumor growth *in vivo* by repressing cell proliferation and migration.

### MIAT knockdown suppressed MMPs expression

**Figure 5:**
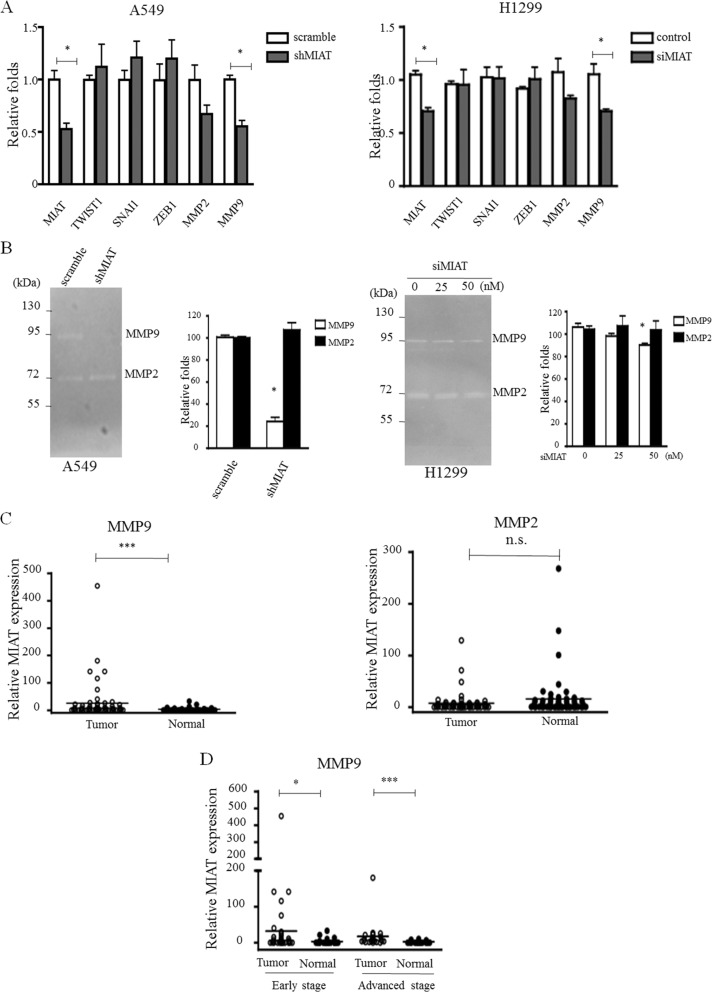
MIAT knockdown represses MMPs activity (**A**) EMT factors and MMPs were selected to elucidate the role of MIAT in tumor progression in A549 (left) and H1299 (right) cells. (**B**) Gelatin zymography was performed to determine the activities of MMP2 and MMP9 in A549 (left) and H1299 (right) cells. Note the clear bands with an apparent molecular weight of < 92 and < 72 kDa, representing gelatinolytic activity of MMP9 and MMP2, respectively. (**C**) MMP9 but not MMP2 was higher expressed in primary human NSCLC compared with adjacent normal tissues (*n* = 60 for each group). (**D**) MMP9 is overexpressed in early and advanced tumor stages. Data are presented as mean ± SD. ^*^p < 0.05, ^**^p < 0.01, ^***^p < 0.001. Statistical analysis was conducted using student *t*-test.

Because the MIAT expression level is correlated with advanced stage and affects cell invasion, we further explored whether MIAT regulated epithelial–mesenchymal transition (EMT) or matrix metallopeptidases (MMPs) expression. The qRT-PCR results revealed that MIAT knockdown has no effect on EMT inducers such as TWIST1, SANI1, and ZEB1; however, MIAT knockdown significantly reduced MMP9 expression (*p* = 0.02) and has a trend of decreasing MMP2 (*p* = 0.07; Figure 5A) in A549 and H1299 cells. We then detected the activities of MMP2 and MMP9 through gelatin zymography. The data revealed that MIAT knockdown could reduce MMP9 expression but has no effects on MMP2 in A549 and H1299 cells (Figure 5B). We also observed that MMP9 but not MMP2 was overexpressed in tumor parts (mean dCT of tumor vs. normal tissue: 1.30 vs. 3.43, *p* < 0.0001; Figure 5C), and that increased MMP9 in early and advanced stages in 60 paired NSCLC tissues was correlated with MIAT (Figure 5D). These results indicated that MMP9 might be a downstream gene that was regulated by MIAT to affect NSCLC migration and invasion.

### MIAT was associated with MLL and epigenetically regulates MMP9 activity

**Figure 6:**
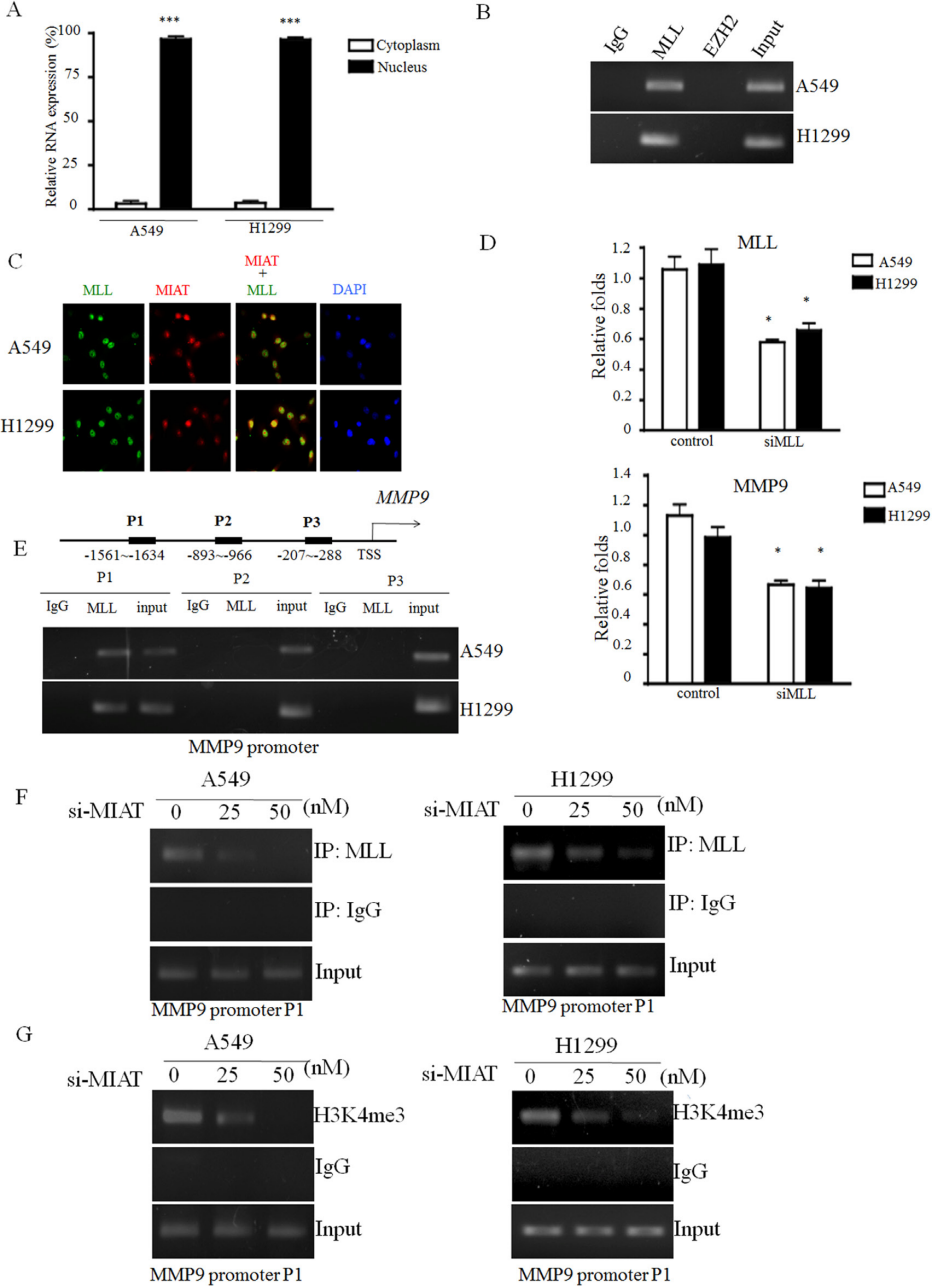
MIAT directly binds with MLL and epigenetically silences MMP9 expression (**A**) Cell fractionation assays were performed to determine the MIAT expression level in the cell cytoplasm and nucleus. (**B**) MIAT RNA level in immunoprecipitates with MLL, EZH2, or IgG was determined using qRTPCR, and the PCR products were loaded on 3% agarose gel for further confirmation. (**C**) Immunofluorescence combined with RNA-FISH assay was performed to examine the localization of MIAT and MLL in the nucleus. Images were observed and analysed using a fluorescence microscope. Red: MIAT; green: MLL; and blue: DAPI stained in nucleus. (**D**) Knockdown of MLL would decrease the expression levels of MLL (upper) and MMP9 (down). (**E**) Three primer sets were designed for ChIP-qPCR experiments on the MLL binding region of the MMP9 promoter. MLL binds to the P1 region of the MMP9 promoter. (**F**, **G**) ChIP-qPCR was used to analyse the MLL occupancy and H3K4me3 status in the MMP9 P1 region after MIAT knockdown. Assays were performed in triplicate. Data are presented as mean ± SD. ^*^p < 0.05, ^***^p < 0.001. Statistical analysis was conducted using student *t*-test.

To investigate the potential mechanism of MIAT in regulating MMP9 in NSCLC cells, we first analysed the distribution of MIAT in cells. The cell fractional data revealed that MIAT was distributed in both the cytoplasm and nucleus; however, the ratio of MIAT in the nucleus was higher than that in the cytoplasm (Figure 6A). Previous studies have reported that lncRNAs could function in cooperation with chromatin-modifying enzymes to promote epigenetic activation or silencing of gene expression. Therefore, we performed the RIP assay to examine whether a physical interaction is present between histone modifiers and MIAT. The results revealed that MIAT could interact with H3K4 methyltransferase MLLs (activator complex) but not EZH2 (enhancer of zeste homolog 2 in polycomb group repressive complex) in A549 and H1299 cells (Figure 6B). We also performed an immunostaining combined with RNA-FISH experiment and observed that MIAT could colocalize with MLL in the nucleus (Figure 6C). To elucidate whether MLL could regulate MMP9 activity, we knockdown MLL expression in A549 and H1299 cells. inthen transfected 50 nM of siMLL into A549 and H1299 to elucidate whether MLL could affect the expression levels of MMP9. The data showed that the expressions of MLL and MMP9 were been silenced (Figure 6D). Therefore, to investigate whether MLL is involved in the regulation of MIAT on MMP9, we first designed three sets of primers of the MMP9 promoter region and found that MLL was bound to the sequences within 1634 bp upstream of the transcript start site (Figure 6E). To further address whether MIAT regulated MMP9 through MLL enrichment, we performed ChIP in the A549 and H1299 cell lines with MIAT knockdown. The results revealed that MIAT silencing reduced the binding activity of MLL and the status of H3K4me3 with the MMP9 promoters in A549 and H1299 cells (Figure 6F and 6G). These results indicated that MIAT interacted with MLL in the nucleus and was essential for MLL to regulate MMP9 activity through epigenetic regulation.

## DISCUSSION

Many studies have reported that lncRNA dysregulation is associated with pathological and physiological processes in different human diseases. The expression levels of lncRNA are also associated with cancer development and progression, including NSCLC [25–27]. For example, lncRNA BANCR functions as a tumor suppressor in NSCLC [28], whereas HOTAIR and MALAT1 promote oncogenic functions in NSCLC [29, 30]. However, the roles of lncRNAs in NSCLC tumorigenesis are still unknown.

MIAT was first identified as a candidate gene for myocardial infarction [16]; it is abundantly expressed in the nervous system [31] and retinal tissue [32]. Recent studies have reported that MIAT participates in chronic lymphocytic leukaemias progression [23] and prostate cancer formation [24]; however, the underlying mechanism of MIAT in tumorigenesis remains unclear. We speculated that MIAT might be involved in NSCLC progression. In the NSCLC cohort, we observed that MIAT was upregulated in lung cancer tissues compared with the non-tumor tissues. In addition, MIAT expression level was significantly different in the advanced tumor stage. Because treatment failure and the poor prognosis of lung cancer are due to high metastasis and invasion, we further identified the biological role of MIAT in tumor progression. A loss-of-function assay demonstrated that MIAT silencing impaired cell proliferation, migration, and invasion *in vitro* and inhibited tumor formation *in vivo*.

To further elucidate the molecular mechanism through which MIAT contributes to invasion and metastasis in NSCLC, we investigated potential target proteins involved in cell motility and matrix invasion. During tumor metastasis, cancer cells lose their polarity and intercellular adhesions and then get invasive characteristics of mesenchymal cells through EMTs [33]. MMPs could degrade the proteins of the extracellular matrix and basement membrane to promote tumor metastasis [34]. Therefore, we determined whether any interaction was present between EMTs, MMPs, and MIAT. MIAT silencing had no effects on EMT transcriptional factors (TWIST1, SNAI1, and ZEB1), but repressed MMP2 and MMP9 expressions in A549 cells. We further determined the expression levels of MMP2 and MMP9 in our NSCLC cohort; the results revealed that the expression level of MMP9 but not MMP2 was correlated with MIAT to express highly in tumor tissue and advanced stage (Figure 1A, 1B and Figure 5C, 5D). MMP9 is a member of the MMP family. MMP9 is overexpressed in colorectal and lung tumor and is related to metastasis and invasion in the breast, upper urinary tract, and oral squamous cell tumor [35–38]. Our findings demonstrated that MIAT-mediated NSCLC cell migration, invasion, and metastasis suppression through MMP9.

**Figure 7:**
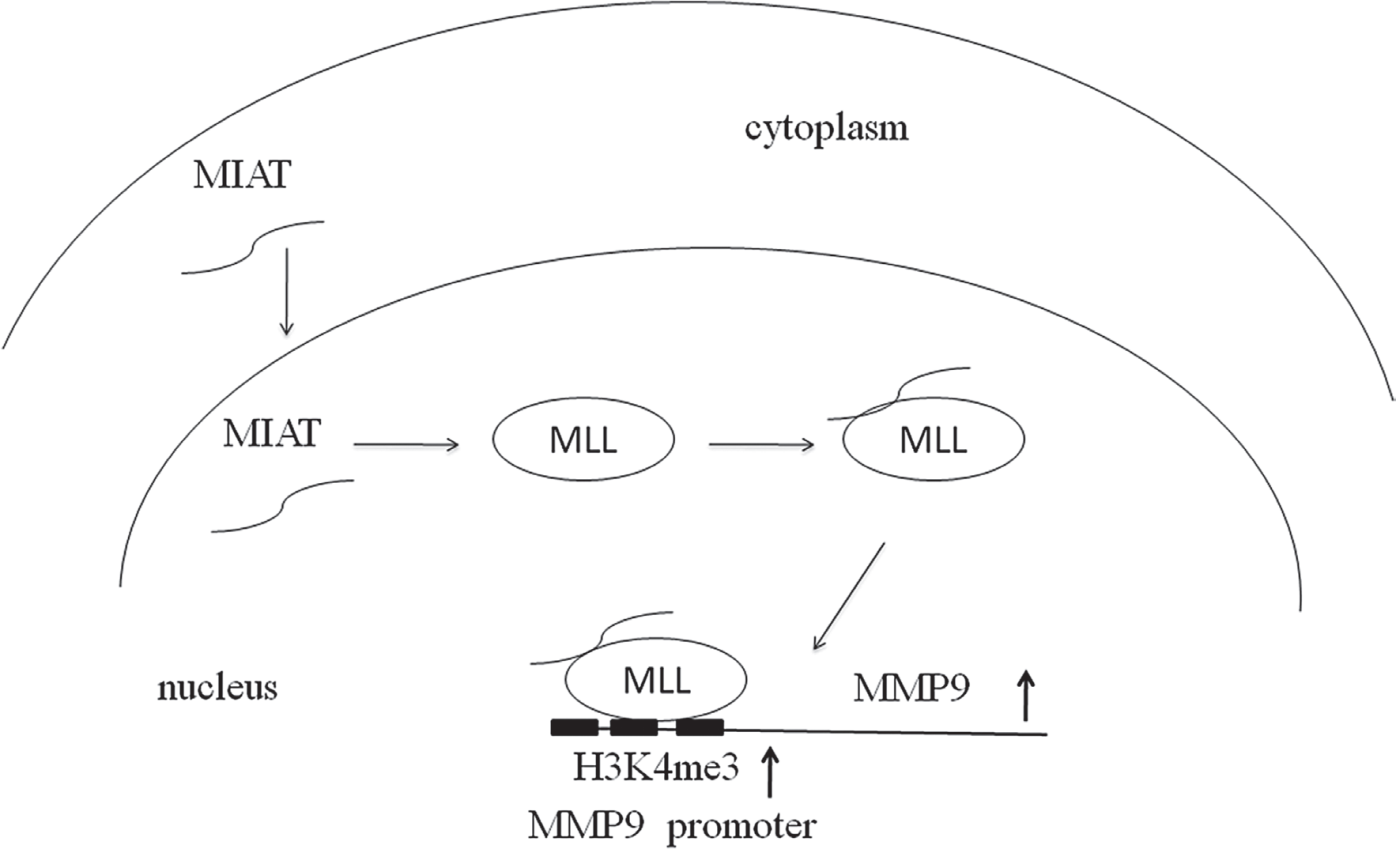
Hypothesis of the mechanism of MIAT on NSCLC MIAT could locate to the nucleus and interact with MLL to epigenetically regulate the status of H3K4me on the promoter of MMP9.

Although several recent studies have indicated that MMP9 expression could also be regulated by some lncRNAs, such as BCRYN1 and MVIH [39, 40], the regulation mechanism remains unclear. Generally, lncRNAs regulate cancer cell phenotypes by interacting with specific RNA binding proteins and resulting in gene activation or repression through chromosome reprogramming, DNA methylation, RNA decay, and histone protein modification [41, 42]. Our RNA-IP experiment demonstrated that MIAT could interact with the histone methyltransferase MLL. MLL is a histone methyltransferase that mediates histone H3 lysine 4 and can catalyze H3K4 monomethylation, dimethylation, and trimethylation [43]. Abnormal methylation of H3K4 frequently occurs in cancer progression. A recent study also revealed that decreased H3K4me3 modification at the MMP9 promoter reduces MMP9 expression and inhibits tumor cell proliferation [44]. Moreover, lncRNAs regulated gene expression by mediating histone modifiers to the promoter of target genes [45–47]. Therefore, we further validated MIAT and MLL on the MMP9 promoter and found that MLL could bind on the MMP9 promoter and that MIAT silencing reduced the binding of MLL and H3K4me3 level with the MMP9 promoter. The hypothesis of the mechanism of MIAT on NSCLC is presented in Figure 7. These results indicated that MIAT might play crucial roles in the MLL-mediated activation of oncogenes in NSCLC.

In conclusion, our results clarified that MIAT was upregulated in NSCLC tumor tissues and was correlated with tumor advanced stage. MIAT knockdown inhibited cell proliferation, migration, and invasion in A549 and H1299 cells and inhibited tumorigenesis *in vivo.* Furthermore, MIAT-mediated oncogenic effects are partially due to the epigenetic silencing of MMP9 through the direct binding of MIAT with MLL. These findings indicate a role of MIAT-dependent histone H3K4 methylation in MMP9 transactivation and lung carcinogenesis and reinforce the notion that targeting the lung cancer epigenome may yield novel therapeutic solutions.

## MATERIALS AND METHODS

### Patients and tissue samples

Sixty paired NSCLC tissues (30 paired of adenocarcinoma and 30 paired of squamous) and adjacent non-tumor tissues from patients (37 males, 23 females, mean age 63.0, SD ± 12.6) who received surgical resection of NSCLC between 2006 and 2014 were obtained from the Bio-Bank of China Medical University Hospital (CMUH) after approval from CMUH's Institutional Research Ethics Committee (CMUH103-REC2-140), according to the Declaration of Helsinki guideline. None of the patients had received chemotherapy or radiotherapy prior to surgery. All surgical specimens were snap-frozen and stored in liquid nitrogen immediately after resection until total RNA extraction. All tumor and paired non-tumor tissues were confirmed by experienced pathologists, and the pathological stage, grade, and nodal status of the tissues were provided. Clinical and pathological characteristics were also collected for each patient. Informed written consent was obtained from all patients in this study.

### Cell culture and transfection

A549 and H1299 cell lines were respectively cultured in DMEM and RPMI supplemented with 10% fetal bovine serum (10% FBS, Gibco), 100 U/ml penicillin, and 100 mg/ml streptomycin in humidified air at 37°C with 5% CO_2_. A549 and H1299 cells were transfected with various dosages of siRNA or negative control by using RNAimax Lipofectamine (Invitrogen, USA) according to the manufacturer's instructions. The siRNA oligonucleotides were synthesized by MDBio, Inc. The siRNA sequences of MIAT were as follows: 5′-ACUUCUUCGUAUGUUCGGCTT-3′ and a negative control (a scrambled matched %GC oligonucleotide). The siRNA sequences of MLL were as follows: 5′-GCUCUUUCCUAUUGGAUAUTT-3′ and a negative control (a scrambled matched %GC oligonucleotide).

### Subcellular fractionation, total RNA extraction, and qRT-PCR analysis

The separation of the nuclear and cytosolic fractions of the A549 and H1299 cell lines was performed according to the protocol of the PARIS Kit (Life Technologies, Carlsbad, CA, USA) Total RNA was extracted from tissues or cultured cells with TRIzol reagent (Life Technologies, Scotland, UK, USA) according to the supplier's instructions. Two micrograms of RNA was reverse-transcribed into cDNA by using a high-capacity cDNA reverse transcriptase kit (Thermo Fisher Scientific-Applied Biosystems, Waltham, MA, USA). Quantitative PCR was performed using the Taqman assay and GAPDH mRNA was employed as an endogenous control for mRNA. Relative expression levels of the target genes were calculated as ratios normalized against GAPDH. The quantification of gene expression was performed by using the 2−ΔΔCt method. All primers were designed and synthesized by Genomics BioSci & Tech, Taipei, Taiwan. The following primer sequences were used: *MMP2*-forward: ccccaaaacggacaaagag, reverse: cttcagcacaaacaggttgc; *MMP9*-forward: cgcagacatcgtcatccagt, reverse: cgcagacatcgtcatccagt; *TWIST1*-forward: ggcatcactatggactttctctatt, reverse: ggccagtttgatcccagtatt; *SANI1-*forward: aggatctccaggctcgaaag, reverse: tcggatgtgcatcttgagg; and *ZEB1-*forward: aactgctgggaggatgacac, reverse: tcctgcttcatctgcctga.

### Cell proliferation assay

Cell proliferation was assessed using a trypan blue exclusion assay. Cells were seeded in 60-mm culture dishes at a density of 1 × 10^5^ cells/dish and incubated for 24 h or 48 h. After incubation, cell number was determined using a trypan blue exclusion test with trypan blue (0.4%) purchased from Sigma Chemical Co.

### Colony formation assay

A total of 10,000 control and siMIAT cells were placed in a 6-well plate and maintained in media containing 10% FBS; the medium was replaced every 3 days. After 14 days, cells were fixed with methanol and stained with 0.1% crystal violet. Viable colonies were manually counted. For each treatment group, wells were measured in triplicate.

### Cell cycle analysis

Transfected A549 and H1299 cells were collected and fixed in 70% ethanol at −20°C overnight. Fixed cells were washed once with PBS and then labelled with propidium iodide (Sigma-Aldrich) in the presence of RNase A (Sigma-Aldrich) and Triton X-100 for 30 min in the dark. Cells were run on a FACSCanto flow cytometer (Becton-Dickinson, FL, NJ, USA). The percentages of the cells within each phase of the cell cycle were analysed using the ModFit LT program.

### Western blot

Whole cell extracts were prepared from A549 and H1299 cells by adding RIPA lysis buffer (150 mM NaCl, 0.1% SDS, 0.5% sodium deoxycholate, 1% NP-40) (Sigma) with complete protease inhibitor cocktails (Sigma). Equal quantities of total protein samples were separated on 10% SDS-PAGE gels and transferred to PVDF membranes. Blots were incubated with primary antibody against Cyclin D3, cdk2 (Abcam) and β-actin (GeneTex) overnight at 4°C. After secondary antibody incubation, the electrochemiluminescence (ECL) kit (EMD Millipore, St. Charles, MO) was used to visualize protein signals. β-actin was used as internal control.

### Wound healing scratch and transwell assays *in vitro*

A wound healing assay was used to assess the ability of cell migration, and appropriate A549 and H1299 cells were seeded into 24-well plates at a density that reached 95%–100% confluence as a monolayer. The monolayer was gently scratched across the centre of the well with a 200-μl plastic tip. The rate of closure was assessed through imaging with an inverted microscope (DMi1; Leica, Wetzlar, Germany). The migration movement throughout the wound area was examined and calculated using the free software ‘TScratch’. For the invasion assay, 24-well transwell chambers with 8 mM pore size polycarbonate membranes were used. Approximately 1 × 10^5^ control or siMIAT cells were seeded into the upper chamber of the insert. After culturing the cells in the upper chamber for 24 h, they were carefully removed, and cells adhering to the underside of the membrane were stained with 0.1% crystal violet solution. The numbers of cells were counted under an inverted microscope (DMi1; Leica, Wetzlar, Germany). For each experimental group, the assay was performed in triplicate.

### *In vivo* tumor formation

BALB/c athymic nude mice (male, 4–6 weeks old) were purchased from National Laboratory Animal Breeding and Research Center, Taiwan. To establish a lung cancer xenograft model, 1 × 10^7^ scramble or shMIAT-A549 cells were suspended in 100 ml PBS and inoculated subcutaneously into the flanks of six nude mice (left: shMIAT; right: scramble). The following shRNA sequence was used for MIAT knockdown: 5′-GATCCCCGGACA GAGAATGCAAATAATTCAAGAG ATTATTTGCATTC TCTGTCCTTTTTA-3′. The tumor size was calculated by measuring length (L) and width (W) with callipers every 3 days. The tumor volumes were calculated using the formula (L × W^2^)/2. All animal experiments were performed in accordance with the guidelines set by the Institutional Animal Care and Use Committee (IACUC) of China Medical University (CMU). All animals were housed in the Laboratory Animal Center of CMU under a 12 h light/dark (08:00/20:00) cycle with free access to food and water. The

mice were sacrificed using CO_2_, and the tissues were subsequently harvested. All breeding and subsequent use of animals in this study, including sacrifice, was approved by the IACUC of CMU. The IACUC approval number was 102-203-N.

### Gelatin zymography assay

Gelatin zymography was performed to determine the activity of MMP2 and MMP9. In brief, the protein in control or siMIAT cell medium were separated in 10% SDS-PAGE containing 3 mg/ml gelatin at 4°C. PAGE was then incubated at 37°C with incubation buffer (50 mM Tris-HCl pH 7.6, 10 mM CaCl_2_.2H_2_O, 50 mM NaCl) for 24 h. Gelatinolytic activities appeared as clear bands after the cells were stained with 0.25% Coomassie brilliant blue R-250.

### RNA immunoprecipitation (RIP)

RNA immunoprecipitation (RIP) was performed using ChIP-IT (Active Motif, Carlsbad, CA, USA) according to the manufacturer's instructions. In brief, endogenous MLL and EZH2 complexes from the whole-cell extract were pulled down using anti-MLL1 (EMD Millipore, St. Charles, MO) and anti-EZH2 (Cell Signaling Technology, Danvers, MA, USA) antibody-coated beads. The beads were washed with wash buffer and eluted with elution buffer. The eluted samples were incubated with 0.5 mg/ml protease K to remove proteins. The isolate from the IP product was further analysed using qRT-PCR. The primers for detecting MIAT expression were as follows: forward: ctggagagggaggcatctaa and reverse: aactcatccccacccacac.

### Immunofluorescence combined with RNA-FISH

Simultaneous protein and mRNA detection using immunofluorescence-combined single-molecule RNA fluorescence *in situ* hybridization (FISH) was performed as described previously [48]. The RNA-FISH probe primer were as follows: forward: tgactccctgaagatctcatcc and reverse: tgctaggaagctgttccagac. The PCR product of MIAT RNA was purified and labelled using Label IT Cy^®^3 nucleic acid labelling kit (Mirus Bio Corp., Madison, WI, USA). In brief, A549 cells were plated on glass coverslips in 6-well culture plates at a density of 10,000 cells/well. Cells were fixed for 10 min in 4% formaldehyde (Thermo Scientific, Rockford, IL, USA) in 1× RNase-free PBS at room temperature. Next, specimens were blocked and permeabilised for 60 min at room temperature in blocking buffer (1× RNase-free PBS, 1% acetylated BSA, 0.3% Triton X-100, and 2 mM vanadyl ribonucleoside complexes). Blocked specimens were incubated with antibodies diluted in blocking buffer. MLL protein was stained with anti-MLL1 (EMD Millipore, St. Charles, MO, USA) and Alexa Fluor 488-conjugated donkey anti-mouse antibody (Jackson ImmunoResearch, West Grove, PA, USA). Incubations with primary antibodies were performed overnight in the dark at 4°C and those with secondary antibodies for 90 min in the dark at room temperature in a humidifying chamber. The RNA-FISH probe were 2192 bps PCR product of MIAT RNA was purified and labelled by Label IT Cy^®^3 nucleic acid labelling kit (Mirus Bio Corp., Madison, WI). After postfixation (10 min in 4% methanol-free formaldehyde in 1× RNase-free PBS at room temperature), the RNA-FISH procedure was performed as described above. Finally, all samples were mounted onto slides in the Vectashield Mounting Medium with DAPI (Vector Laboratories, Burlingame, CA, USA), sealed with nail polish, and imaged using a Leica DMI6000B (AF7000 version) inverted widefield fluorescence microscope (Leica Microsystems, Wetzlar, Germany).

### Chromatin immunoprecipitation (ChIP)

DNA ChIP assay was performed using ChIP-IT (Active Motif, Carlsbad, CA, USA) according to the manufacturer's instructions by using Anti-MLL1 (EMD Millipore, St. Charles, MO, USA), anti-EZH2 (Cell Signaling Technology) antibodies, and IgG. Three sets of primers were designed to amplify the MMP9 promoter region. P1 (−1563 to −1634)-forward: ggagatttggctgcatgg, reverse: gcaggatatgggggaaaataat; P2 (−893 to −966)-forward: cctagcagagcccattcctt, reverse: ccctgacagccttctttgac; and P3 (−207 to −288)-forward: cagtccacccttgtgctctt, reverse: ctaggtgtttgcccacctct.

### Statistical analysis

All experimental data from three independent experiments were analyzed by GraphPad Prism version 5 (GraphPad Software Inc., La Jolla, California, USA) and results were expressed as mean ± SD (standard deviation, SD). The association between relative MIAT RNA expression levels and clinical parameters (age, gender, tumor size, lymph node metastasis and TNM stage) was analyzed using Fisher's exact test. Student *t*-test was conducted to analyze the *in vitro* and *in vivo* assays. *P* < 0.05 was considered to indicate a statistically significant difference.
